# Structural features on quantitative chest computed tomography of patients with maximal mid-expiratory flow impairment in a normal lung function population

**DOI:** 10.1186/s12890-023-02380-0

**Published:** 2023-03-15

**Authors:** Yuling Yang, Haiyan Ge, Jinjuan Lu, Xuemei Huang, Kun Wang, Liang Jin, Lin Qi, Ming Li

**Affiliations:** 1 Department of Radiology, Huadong Hospital Affiliated With Fudan University, No. 221 West Yanan Road, Shanghai, 200040 China; 2 Department of Respiratory Medicine, Huadong Hospital Affiliated With Fudan University, No. 221 West Yanan Road, Shanghai, 200040 China

**Keywords:** COPD, Maximal mid-expiratory flow, Quantitative computed tomography

## Abstract

**Background:**

Maximal mid-expiratory flow (MMEF) is an earlier predictor of chronic obstructive pulmonary disease (COPD) development than forced expiratory volume in 1 s (FEV_1_). Changes of lung structure in patients with MMEF impairment only is still not clear. Therefore, this study aimed to investigate the structural features of patients with decreased MMEF by quantitative computed tomography (QCT) and develop a predictive model for predicting patients with reduced MMEF in normal lung function population.

**Methods:**

In this study, 131 patients with normal spirometry results and available volumetric chest CT images were enrolled and divided into the reduced MMEF group (FEV_1_/forced expiratory vital capacity (FEV_1_/FVC) > 0.7, FEV_1_% predictive values (FEV_1_%pred) > 80%, MMEF%pred < 80%, *n* = 52) and the normal MMEF group (FEV_1_/FVC > 0.7, FEV_1_%pred > 80%, MMEF%pred ≥ 80%, *n* = 79). The emphysema, small airway disease and medium-size airway parameters were measured by a commercial software. The differences were investigated in clinical features, spirometrical parameters and QCT parameters between the two groups. A nomogram model was constructed based on the results of the multivariable logistic regression model. Spearman’s correlation coefficients were calculated between QCT measurements and spirometrical parameters.

**Results:**

There were more males in reduced MMEF group than normal group (*P* < 0.05). Lung parenchyma parameter (PRM^Emph^) and airway-related parameters (functional small airway disease (PRM^fSAD^), luminal area of fifth- and sixth- generation airway (LA_5_, LA_6_) were significantly different between the reduced MMEF group and the normal group (20.2 ± 17.4 vs 9.4 ± 6.7, 3.4 ± 3.5 vs 1.9 ± 2.0, 12.2 ± 2.5 vs 13.7 ± 3.4, 7.7 ± 2.4 vs 8.9 ± 2.8, respectively, all *P* < 0.01). After multivariable logistical regression, only sex (odds ratio [OR]: 2.777; 95% confidence interval [CI]:1.123–3.867), PRM^fSAD^ (OR:1.102, 95%CI:1.045–1.162) and LA_6_ (OR:0.650, 95%CI:0.528–0.799) had significant differences between the two groups (*P* < 0.05) and a model incorporating with the three indicators was constructed (area under curve, 0.836). Correlation analysis showed MMEF%pred had mild to moderate correlation with airway-related measurements.

**Conclusion:**

In normal lung function population, patients with reduced MMEF have potential medium-size and small airway changes, and MMEF%pred is significantly associated with airway-related CT parameters. The nomogram incorporating with sex, PRM^fSAD^ and LA_6_ has good predictive value and offers more objective evidences in a group with reduced MMEF.

**Supplementary Information:**

The online version contains supplementary material available at 10.1186/s12890-023-02380-0.

## Background

Chronic obstructive pulmonary disease (COPD) is a type of progressively obstructive lung disease that is characterized by persistent respiratory symptoms and airflow limitation due to airways and alveolar abnormalities [1–3]. Forced expiratory volume in 1 s (FEV_1_) to forced expiratory vital capacity (FVC) of less than 0.7 is the gold standard to confirm the diagnosis of COPD [4], and a decrease in FEV_1_ is considered to be a predictor of COPD development [5]. However, recent studies suggest that maximal mid-expiratory flow (MMEF) values has been shown to occur before a decrease in FEV_1_, and could be an indicator of early disease [6, 7]. MMEF, also referred to as FEF_25-75_, defined by the the American Thoracic Society (ATS) and European Respiratory Society (ERS) as the mean forced expiratory flow between the 25% and 75% of the FVC [8], is widely accepted as a measure of small airways (diameter < 2 mm) obstruction. A 10-year follow-up study reported that MMEF is an earlier predictor of COPD development than FEV_1_ in normal lung function population [9]. Furthermore, a recent work reveals smokers with a decreased MMEF are approximately eight times more likely to develop COPD than patients with a normal MMEF, despite having FEV_1_% predicted (FEV_1_%pred) > 80% [10]. Indeed, MMEF rates have been considered a sensitive way to detect early stages of obstructive airway disease since the 1970s [11]. But MMEF has recently been found to be associated not only with small airway abnormalities, but also with lung parenchyma changes. Small airways abnormalities and destruction of lung parenchyma (emphysema) are major pathological hallmarks of COPD, which would contribute to airflow limitation via distinct mechanisms [12]. However, MMEF rates could not provide anatomic localization and quantitative characteristics of either of the two major disease. Computed tomography (CT) can serve as a complementary tool for pulmonary function test (PFT) by providing anatomic localization, differentiating airway disease from emphysema and characterizing emphysema subtypes [13]. Small airway disease can be inferred from an indirect measure of air trapping on CT images, and emphysema are the voxels with CT attenuation less than -950 Hounsfield unit (HU) on inspiration.

Therefore, the purpose of this study was to investigate the imaging features of patients with MMEF abnormalities and develop a predictive model for predicting patients with abnormal MMEF by CT images.

## Methods

### Study subjects

**Fig. 1:**
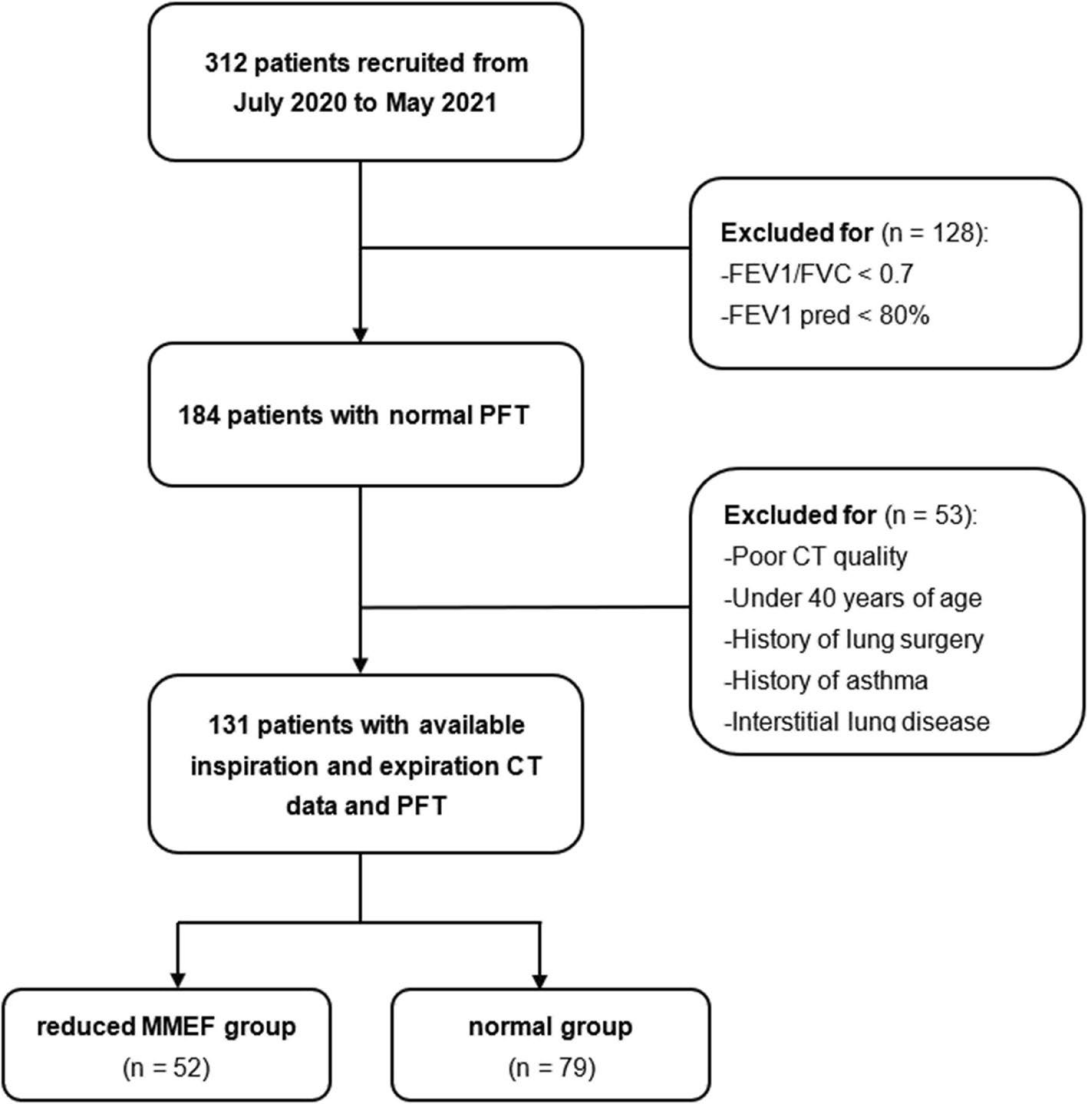
Flowchart of this study population

This prospective study was approved by the Ethics Committee of Huadong Hospital Affiliated to Fudan University (No. 2021K018), and informed consents were obtained from all patients. The study is based on a retrospective interpretation of prospectively acquired data. From July 2020 to May 2021, 312 participants who came for annual health screening without respiratory symptoms at our hospital were enrolled in the study. Subjects who met any of following criteria were considered for inclusion from the analysis with normal lung function results (FEV_1_/FVC > 0.7 and FEV_1_%pred > 80%), age > 40 years, no history of asthma or interstitial lung disease, and no history of lung surgery. Forty-one out of 184 eligible subjects were excluded because of incomplete data or poor CT scan quality (CT image quality evaluation detailed in CT examination). Finally, we remain 131 patients with normal PFT results and grouped them according to the MMEF predictive values (Fig. 1).

### Clinical parameters

Clinical parameters, included the body-mass index (BMI), age, gender and smoking status, were collected to estimate differences between the two groups. BMI was calculated by dividing weight by height squared (kg/m^2^). Smoking status was classified as current-smoker, former-smoker and never-smoker according to the total number of smoked cigarettes, in accordance with previous studies [14, 15]. Current-smokers were considered to have smoked at least 100 cigarettes during their lifetime and currently smoked on a few days or every day. Former-smoker had smoked at least 100 cigarettes, but is current non-smoker. Never-smoker had never smoked or smoked less than 100 cigarettes in their life.

### PFTs

All spirometry tests were performed within 1 month before or after CT scanning. The procedures were conducted on a Jaeger MasterScreen Pro lung function system (Jaeger Ltd, Hochberg, Germany) according to the ATS/ERS standardization of spirometry [16]. Baseline FEV_1_, FVC, FEV_1_/FVC, MMEF, residual volume (RV), total lung capacity (TLC), and RV/TCL values were obtained for the study. FEV_1_ and FVC were expressed as percentage of normal predicted values. In current study, to reduce the variability of MMEF and the influence of aging-related decline in lung function, MMEF was expressed as % predicted. Below 80% of predicted value was considered as MMEF abnormalities, as reported in previous studies [17, 18].

### CT scanning

Prior to the examination, all patients had been previously instructed on how to perform the respiratory maneuvers while lying in the CT scanner acquisition bed. Patients underwent volumetric thin-section chest CT at both full inspiration and full expiration in the supine position. Scans were performed with a dual-source CT system (Somatom Definition Flash, Siemens Healthcare, Forchheim, Germany) in the caudocranial direction using the following parameters: tube voltage, 140 kVp; effective tube current, 100 mAs; slice thickness, 0.6 mm; picth, 1.0; and gantry rotation time, 0.5 s. The acquired data from the thoracic inlet to the lung base were reconstructed using B30f kernel. All the images were subjectively general evaluated by a radiologist (with 20 years of experience) according to the European Guidelines on Quality Criteria for Computed Tomography [19]. Diagnostic acceptability was evaluated with a four-point scale (1 = fully acceptable, 2 = probable acceptable, 3 = acceptable only in limited conditions, 4 = diagnostically unacceptable). Image noise was evaluated with a three-point scale (1 = too little or less than usual noise, 2 = acceptable noise, 3 = excessive noise). Images with diagnostic acceptability or image noise scores of 3 and above will be excluded for software analysis.

### Quantitative image analysis

**Fig. 2:**
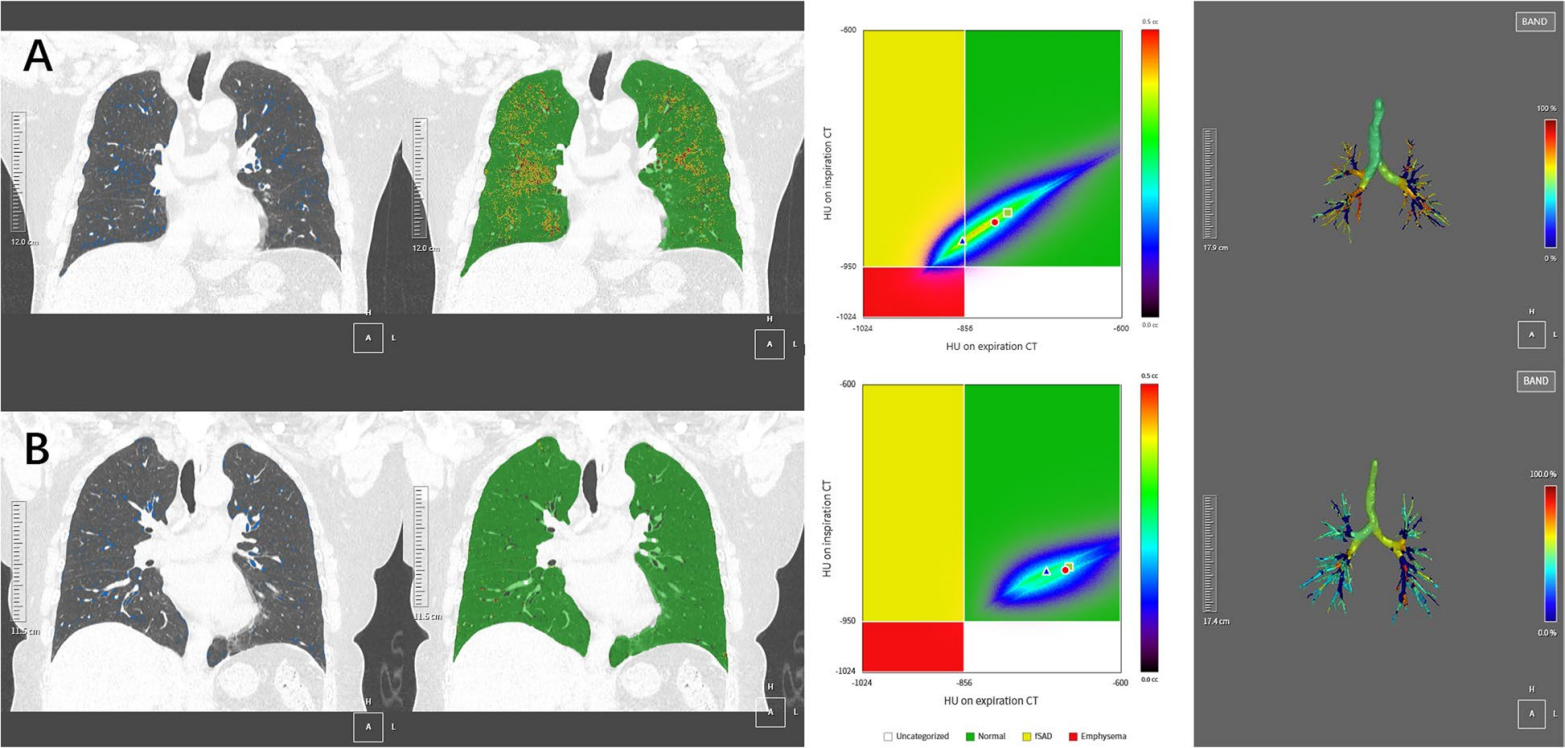
Images of lung parameters in two patients. Notes: The first column shows the distribution of emphysema (displayed in blue). The second and third columns illustrate the distribution of voxels corresponding to PRM class (PRM^fSAD^ voxels in yellow, PRM^Emph^ voxels in red, and normal voxels in green). The last column shows the tracheobronchial tree generated by three-dimensional reconstruction; **a** A 61-year old male with reduced MMEF (FEV_1_/FVC = 0.84, FEV_1_%pred = 91.6%, MMEF%pred = 67.0%). The emphysema index is 5%. PRM^fSAD^, PRM^Emph^ and PRM^Normal^ are 16%, 4% and 78% respectively. The luminal area of the fifth- and sixth- bronchi are 10.4 mm^2^ and 5.2 mm^2^ respectively; **b** A 58-year old female with normal pulmonary function (FEV_1_/FVC = 0.96, FEV_1_%pred = 99.0%, MMEF%pred = 121.1%). The emphysema index is 3%. PRM^fSAD^, PRM^Emph^ and PRM^Normal^ are 1%, 1% and 97% respectively. The luminal area of the fifth- and sixth- bronchi are 14.3 mm^2^ and 6.6 mm^2^ respectively. PRM, Parametric response mapping; PRM^Emph^, emphysema by PRM; RPM^fSAD^, functional small airway disease; PRM^Normal^, normal lung parenchyma by PRM

All CT images were evaluated using a commercial software (Aview, Coreline Soft, Seoul, Korea). Both lungs as well as each lung lobe were automatically segmented with manual edits as necessary by a professional radiographer. Emphysema was quantitated using the percentage of low-attenuation units less than -950HU at maximal inspiration, and recorded as emphysema index (EI). Small airway disease was quantified by Parametric response mapping (PRM) which utilizes image matching by deformed co-registered inspiratory and expiratory CT images and classifies based on a voxel-by-voxel comparison of lung attenuation changes: (1) functional small airway disease (PRM^fSAD^), are voxels greater than -950HU on inspiratory CT and less than -856HU on expiratory CT, (2) emphysema (PRM^Emph^), are voxels less than -950HU on inspiratory CT and less than -856HU on expiratory CT, (3) normal (PRM^Normal^), are voxels greater than -950HU on inspiratory CT and greater than -856HU on expiratory CT (Fig. 2). PRM data were expressed as percentage of total lung volume. Airways were automatically segmented and airway level was detected, and the airway parameters were recorded in medium-size airways, including Pi10, wall thickness (WT), airway wall area percent (%WA) and luminal area (LA). Pi 10 was the square root of the wall area of a hypothetical airway with 10 mm internal perimeter, which was a useful measure of airway wall thickness obtained by calculating a regression line that was plotted from the square root of the wall area of internal perimeters of multiple airways at different locations [20–22]. Wall thickness, airway wall area percent and luminal area were calculated from the average of whole lung fifth and sixth generation whole lung bronchial values. The software magnified the images tenfold to automatically detect airways lumens and to measure wall area and luminal area. The wall thickness was calculated using full-width-half-maximum (FWHM) measurement algorithm by detecting the inner and outer boundaries of the airway wall, as early study described [23]. Airway wall area percent was calculated as follows: WA / (WA + LA) *100.

Finally, the quantitative computed tomography (QCT) measurements used in this study included EI, PRM (PRM^fSAD^, PRM^Emph^, PRM^Normal^), Pi10, mean %WA of the 5th and 6th generation bronchi, mean WT of the 5th and 6th generation bronchi and LA of the 5th and 6th generation bronchi.

### Statistical analysis

All statistical analyses were performed using R software (version 3.5.1; http://www.Rproject) and MedCalc Software (version 16.8.4, http://www.medcalc.org). Parametric data were expressed as mean ± standard deviation, and nonparametric data were expressed as numbers and percentages. Student’s t test was used for continuous variables, Pearson’s chi-squared test and Fisher’s exact test was used for categorical variables. Clinical and imaging variables with a *P* value < 0.1 on univariable analysis were selected as input variables for multivariable logistic regression analysis with a backward stepwise selection mode. A nomogram model was constructed based on the results of the multivariable logistic regression model to make the result of this research feasible to clinicians. Calibration curve was plotted to assess the fitting efficiency of the nomogram.

The receiver operating characteristic (ROC) curve and area under the ROC curve (AUC) were used to evaluate the predictive nomogram. Spearman’s correlation coefficients were calculated between QCT measurements and pulmonary function parameters. Correlation coefficients were interpreted according to the following categories: *r* < 0.3, mild correlation; 0.3 < *r* < 0.7, moderate correlation; and *r* > 0.7, strong correlation. Two-sided P-value less than 0.05 was considered statistically significant.

## Results

### Patients’ characteristics

The final cohort compromised 131 subjects with normal pulmonary function test and evaluable quantitative CT, and patients were divided into two groups based on the predictive MMEF%: 52 cases of the reduced MMEF group (FEV_1_/FVC > 0.7, FEV_1_%pred > 80%, MMEF%pred < 80%), 79 cases of the normal MMEF group (FEV_1_/FVC > 0.7, FEV_1_%pred > 80%, MMEF%pred ≥ 80%). The clinical characteristics, pulmonary function results and QCT parameters are presented in Table 1. There was a greater proportion of males in reduced MMEF group than that in the normal group (63.5% vs 41.8%, *P* = 0.015). But the smoking status was not significantly different between the two groups (*P* = 0.085). Compared with PFT results between the two groups, FEV_1_%pred values in the reduced MMEF group were significantly lower than those in the normal MMEF group, while there was no significant difference in actual measurements of FEV_1_ between the two groups. Although FEV_1_/FVC was significantly different between the two groups, these values were all within the normal ranges. There was no significant difference in EI value between the reduced MMEF group and normal group (5.7 ± 5.5 vs 4.0 ± 4.1, *P* = 0.051). The average values of PRM^fSAD^, PRM^Emph^ and PRM^Normal^ were significantly different between the reduced MMEF group and the normal group (20.2 ± 17.4 vs 9.4 ± 6.7, 3.4 ± 3.5 vs 1.9 ± 2.0, 73.8 ± 19.7 vs 85.3 ± 10.0, respectively, all *P* < 0.05). The average the luminal area of fifth generation bronchi (LA_5_) and the luminal area of sixth generation bronchi (LA_6_) value were significantly smaller in reduced MMEF group than that in normal MMEF group (12.2 ± 2.5 vs 13.7 ± 3.4, 6.9 ± 2.0 vs 8.9 ± 2.8, respectively, all *P* < 0.05). Pi10, airway wall thickness and wall area percent showed no significant difference between reduced MMEF group and normal MMEF group (all *P* > 0.05).

**Table 1:** Demographics, lung functions and imaging parameters of patients

Parameters	reduced MMEF group (*n* = 52)	normal MMEF group (*n* = 79)	*P* value
Age, years	61.7 ± 9.2	60.6 ± 11.4	0.547
Sex, male (%)	33(63.5%)	33(41.8%)	0.015*
Smoking status			0.081
never-smoker (%)	27(51.9%)	55(69.6%)	
current-smoker (%)	22(42.3%)	19(24.1%)	
Former-smoker (%)	3(5.8%)	5(6.3%)	
BMI	23.7 ± 3.1	24.5 ± 3.3	0.169
FEV_1_ (L)	2.2 ± 0.5	2.4 ± 0.6	0.164
FEV_1_ (% predicted)	92.2 ± 8.2	106.5 ± 14.4	< 0.001***
FVC (L)	2.7 ± 0.6	2.6 ± 0.6	0.411
FVC (% predicted)	89.2 ± 11.0	93.9 ± 14.7	0.053
FEV_1_/FVC	83.2 ± 7.5	91.3 ± 6.0	< 0.001***
MMEF (% predicted)	65.5 ± 11.7	110.3 ± 21.9	< 0.001***
RV (L)	2.6 ± 2.4	2.2 ± 0.5	0.130
TCL (L)	4.7 ± 0.9	4.7 ± 0.9	0.768
RV/TLC (%)	48.4 ± 6.3	47.0 ± 6.9	0.239
EI	5.7 ± 5.5	4.0 ± 4.1	0.051
PRM			
PRM^fSAD^	20.2 ± 17.4	9.4 ± 6.7	< 0.001***
PRM^Emph^	3.4 ± 3.5	1.9 ± 2.0	0.006**
PRM^Normal^	73.8 ± 19.7	85.3 ± 10.0	< 0.001***
Pi10	3.3 ± 0.6	3.1 ± 0.7	0.089
WT_5_	1.4 ± 0.3	1.3 ± 0.3	0.427
%WA_5_	61.0 ± 8.8	59.8 ± 9.6	0.468
LA_5_	12.2 ± 2.5	13.7 ± 3.4	0.008**
WT_6_	0.9 ± 0.3	0.8 ± 0.2	0.187
%WA_6_	55.3 ± 9.6	53.1 ± 10.3	0.239
LA_6_	6.9 ± 2.0	8.9 ± 2.8	< 0.001***

Data are expressed as number (percentage) or median ± standard deviation. * P < 0.05, ** P < 0.01, *** P < 0.001

*Abbreviations: BMI* body-mass index, *FEV*_*1*_ forced expiratory volume in 1 s, *FVC* forced vital capacity, *MMEF* maximal mid-expiratory flow, *RV* residual volume, *TCL* total lung capacity, *EI* emphysema index, *LA* luminal area, *Pi10* the square root of the wall area of a hypothetical airway with 10 mm internal perimeter, *PRM* parametric response mapping, *PRM*^*Emph*^ emphysema by PRM, *PRM*^*fSAD*^ functional small airway disease, *PRM*^*Normal*^ normal lung parenchyma by PRM, *%WA* airway wall area percent, *WT* wall thickness

#### Association of CT parameters with spirometrical results

The quantitative CT parameters with significant differences were selected for the correlation analysis to evaluate the pulmonary function parameters, and the results are presented in Supplementary Table S1. Most of the quantitative CT parameters has significant correlation with PFT results. MMEF %pred has significant correlation with small airway parameter (PRM^fSAD^), medium airway parameters (LA_5_, LA_6_), with stronger correlation with LA_6_ than other QCT parameters (*r* = 0.338, *P* < 0.001). FEV_1_%pred was significantly correlated with the luminal area of the 6th generation bronchi only and not correlated with luminal area of the 5th generation bronchi.

#### Nomogram and calibration curve

**Fig. 3:**
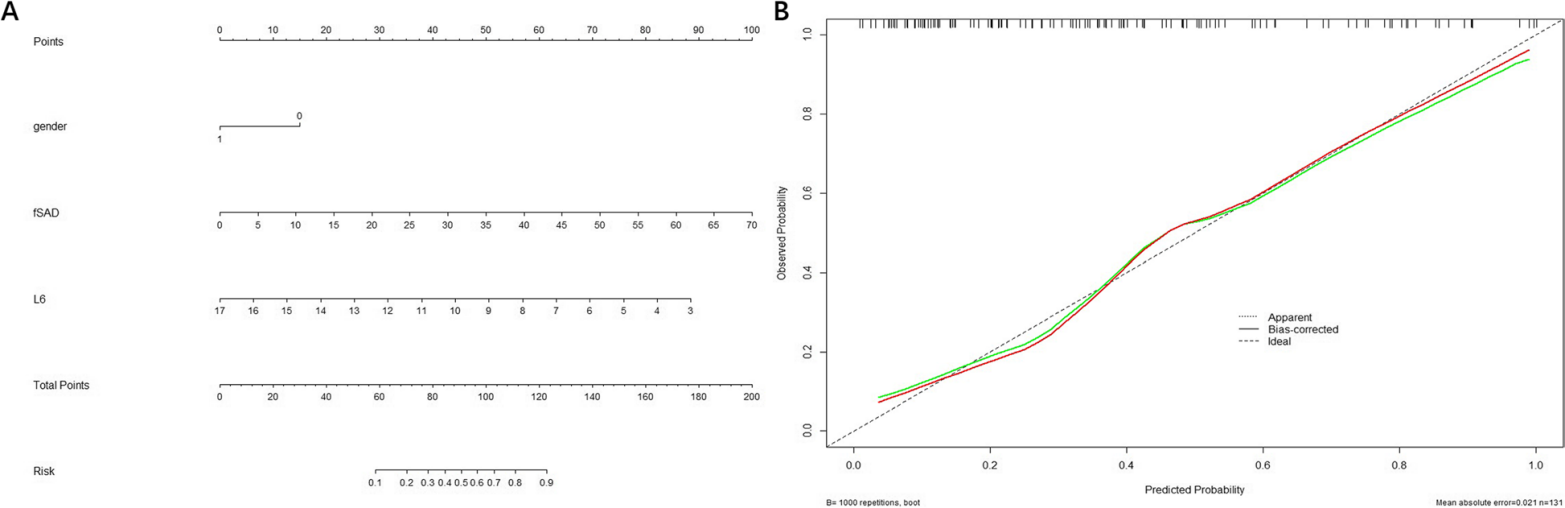
Construction of the nomogram. Notes: 1 for female, 0 for male. **a** Nomogram predicting MMEF reduction; **b** Calibration curves for the nomogram. fSAD, functional small airway disease; L_6_, luminal area of 6th-genneration bronchi

**Fig. 4:**
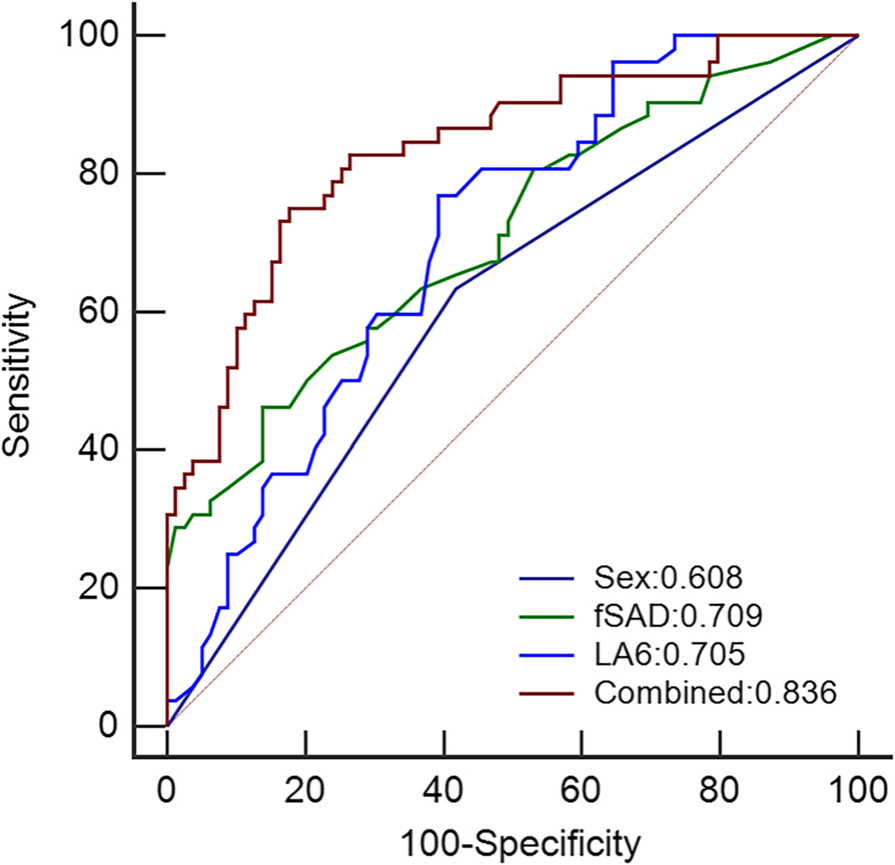
The ROC curves of the nomogram and the single variables. Notes: Combined was the model incorporating with sex, fSAD and LA_6_. fSAD, functional small airway disease; LA_6_, luminal area of 6th-genneration bronchi

Sex, smoking status, EI, PRM parameters, Pi10, LA_5_ and LA_6_ were used as input variables for the multivariable regression analysis. Sex, PRM^fSAD^ and LA_6_ remained significantly different between the two groups after multivariable regression analysis, and other parameters showed no significance (all *P* > 0.05). Subsequently, the three parameters were selected to establish the model (Table 2), and nomogram of the model shows in Fig. 3A. Calibration plots were used to visualize the performance of the nomogram (Fig. 3B). To demonstrate the clinical advantages of the nomogram, we compared the receiver operating characteristic (ROC) curves of the single variables against the model (Fig. 4). It was revealed that the area under the curve (AUC) values of sex, PRM^fSAD^ and LA_6_ were 0.608 (95% confidence interval (CI):0.519–0.693), 0.709 (95% CI: 0.624–0.785) and 0.705 (95% CI: 0.619–0.782) respectively, while the AUC value of nomogram was 0.836 (95% CI: 0.762–0.895). DeLong’s test was used to compare the difference in the ROC curves and showed that the nomogram performed better than sex, PRM^fSAD^ and LA_6_ alone (all *P* < 0.05).

**Table 2:** Logistic regression analysis for prediction of patients with decreased MMEF

Variables	OR (95% CI)	*P* value
Sex	2.777 (1.123, 3.867)	0.027
PRM^fSAD^	1.102 (1.045, 1.162)	< 0.001
LA_6_	0.650 (0.528, 0.799)	< 0.001

*Abbreviations: CI* Confidence Interval, *OR* Odds Ratio, *LA*_*6*_ luminal area 6th generation of bronchi, *RPM*^*fSAD*^, functional small airway disease, *MMEF* maximal mid-expiratory flow

## Discussion

In the present study, we investigated the CT image characteristics of patients with decreased MMEF in a normal lung function population, analyzed the correlations between the quantitative CT parameters and lung function parameters, and developed a predictive model for predicting patients with reduced MMEF.

A study found that patients with reduced MMEF had a greater proportion of males than normal subjects but it is not clear whether sex is a risk factor for MMEF reduction [9]. Stockly et al. [18] analyzed a cohort of α1-antitrypsin deficiency (AATD) non-smokers and found that most of the patients in whom MMEF decreased were females. The population of the present study differs from that of the Stockly et al.’s study and includes both smokers and nonsmokers without AATD. In this study, there was a significant difference in sex between reduced MMEF group and normal group and sex was found to be a predictor for distinguishing between the two groups. Additionally, no significant difference in EI was found between the reduced MMEF group and normal group, while difference in PRM^Emph^ was found between the two groups. Both the emphysema parameters, EI and PRM^Emph^, which reflect pulmonary parenchyma destruction, were less than 6%, indicates that there was no emphysema in the reduced MMEF group, since the Fleischner Society guidelines define emphysema as present only when pixels less than -950HU at quantitative CT are more than 6% [24]. This result is in line with previous studies [18, 25]. Besides, Burgel [26] suggested the key factor leading to airflow limitation in early COPD is small airway disease rather than emphysema. However, changes in the small airways cannot be visualized with current CT capabilities. Investigators have explored measuring air trapping to quantify small airway disease in CT, including measurements of LAA_-__856exp_ (lung voxels with CT attenuation less than or equal to -856 HU measured on expiratory CT scan), E/I (ratio of mean lung attenuation on expiratory and inspiratory scans) and PRM [27–29]. However, E/I is not able to provide spatial information related to disease distribution and LAA_-__856exp_ do not discriminate between air trapping as a result from emphysema or air trapping as a result from small airway disease. PRM uses digitally co-registered inspiratory and expiratory CT scans to compare individual voxel lung attenuation changes allowing differentiation of emphysematous from non-emphysematous air trapping within the lung parenchyma [30]. This non-emphysematous air trapping could reflect the functional changes of small airways, so it is called "functional small airway disease (PRM^fSAD^)". Vasilescu and colleagues [31] performed CT and micro-CT examinations of lung tissue and have confirmed the PRM^fSAD^ correlated well with pathological small airway disease. Lu et al. [32] showed that among patient with abnormal lung function (FEV_1_predicted % < 80% or FEV_1_/FVC < 0.70), air trapping on CT images in patients with reduced MMEF was more severe in than subjects without MMEF reduction, a result is similar to that of Arakawa and colleagues [33]. However, in the studies by Lu et al. and Arakawa et al., they all used air trapping as a proxy for small airway disease, which may exaggerate the extent of small airway disease in patients with decreased MMEF owing to the effects of emphysematous air trapping and overestimate the correlation between MMEF and small airways overestimate the correlation between MMEF and small airways. In this paper, small airway disease was assessed by using PRM^fSAD^ from dual gas phase CT image analysis, which may be more helpful in accurately evaluating the relationship between MMEF impairment and small airway disease. Additionally, the current study found that functional small airway disease, which is a prominent predictor of the model, was significantly higher in MMEF impairment group than in normal group. PRM parameters have been found to be strongly correlated with MMEF%pred, but PRM^fSAD^ was more strongly associated with MMEF%pred than the other two PRM parameters. The result is similar to a smoker study from SPIROMICS cohort [34] in which an increase in PRM^fSAD^ is significantly associated with lower MMEF%pred even adjusted FEV_1_%pred and FVC%pred. Therefore, our analysis supports that patients with MMEF%pred reduction have no destruction to lung parenchyma but changes in small airways, and PRM^fSAD^ also has a good differentiation for normal and reduced MMEF.

In addition, the medium-size airway measurement parameters also need to be noted. We found no evidence for differences in airway wall thickness measurements, including Pi10 and WT, between subjects with and without MMEF reduction. Nambu et al. [35] concluded that CT images of patients with reduced MMEF showed thicker airway walls, which is inconsistent with our results. However, their research included all COPD patients and not adjusted for the effects of airway remodeling due to other apparently abnormal spirometrical parameters. Airway wall thickening could be due to airflow obstruction [36], whereas none of the patients enrolled in this study had evidence of respiratory airflow obstruction. Qin and colleagues [25] studies smokers and found there was no difference in percentage of the third-, fifth- and ninth-generation bronchial wall area between normal subjects and patients with MMEF reduction. Similarly, in the study, through the analysis of normal and decreased MMEF populations, we found no significant difference in fifth- and sixth-generation bronchial wall area percent between the two groups. Nevertheless, the CT scan lumen area in fifth- and sixth-generation airways was significantly reduced in patients with reduced MMEF compared with health controls and reduced luminal area in fifth- and sixth-generation airway was associated with a reduced MMEF%pred. Previous study of asthmatics has also shown that the smaller airway lumen in patient with decreased MMEF%pred, which the researchers believe that may the mucus accumulation reduce the airway luminal area [37], the but it needs to be further studied.

In the current study, we performed a quantitative analysis on patients with MMEF reduction, and found no lung parenchyma destruction but potential medium and small airway changes in these patients compared to subjects without decrease MMEF. Furthermore, a predictive model was developed based on sex, PRM^fSAD^ and LA_6_ for predicting patients with MMEF impairment with AUC of 0.836. Besides, correlation analysis revealed a strong correlation between MMEF and airway-related CT parameters. MMEF impairment has been commonly reported to be associated with some respiratory cdisease, such as allergic rhinitis, asthma and bronchiolitis obliterans [38–40], and other systemic disease associated with decreased MMEF has been rarely reported [41, 42]. This article only discusses the structural lung abnormalities associated with decreased MMEF, but does not explore whether other systemic abnormalities are also correlated with decreased MMEF, which will require further study in the future.

This study also has some limitations. First, parametric response mapping analysis needs dual-phase CT scanning which increase the expose of radiation dose. Second, due to the limited resolution of CT, the wall thickness of airway with diameter less than 2 mm may be overestimated and luminal area can be underestimated. However, an optical coherence tomography study revealed airways with diameter less than 2 mm located at the seventh (or higher) airway generation [43]. Therefore, resolution has little influence on the evaluation accuracy of direct airway parameters from 5th- and 6th-genneration bronchi in the current study. Third, the correlation between pulmonary function test results and CT parameters are not as strong as previous studies, possibly due to the relatively small sample size. Last, the study is a cross-sectional research about patients with MMEF reduction and it is unclear whether these patients will develop COPD later in life. Further studies will enroll more subjects and follow them longitudinally to observe if they will develop COPD.

## Conclusions

In summary, our study shows that in normal lung function population, patients with reduced MMEF have potential medium-size and small airway changes, and MMEF is significantly associated with airway-related CT parameters. The nomogram incorporating with sex, PRM^fSAD^ and LA_6_ can has good predictive value. The present study offers new insight into the pathologic changes captured by CT scan in a group with reduced MMEF and potentially assists clinicians to take early interventions in these patients.

## Supplementary Information

**Additional file 1.**

## Data Availability

The data that support the findings of this study are available from the corresponding author upon reasonable request.

## Abbreviations

COPD: Chronic obstructive pulmonary disease
FEV_1_: Forced expiratory volume in 1 s
FVC: Forced vital capacity
MMEF: Maximal mid-expiratory flow
PFT: Pulmonary function test
PRM: Parametric response mapping
QCT: Quantitative computed tomography
RV: Residual volume
TLC: Total lung capacity
WA: Wall area
WT: Wall thickness
LA: Luminal area
PRM^Emph^: Emphysema by PRM
PRM^fSAD^: Functional small airway disease
PRM^Normal^: Normal lung parenchyma by PRM
EI: Emphysema index
