# Supplementary material for: Structural features on quantitative chest computed tomography of patients with maximal mid-expiratory flow impairment in a normal lung function population
Source: BMC Pulm Med. 2023 Mar 15;23:86. doi: 10.1186/s12890-023-02380-0 (PMC10015933; doi:10.1186/s12890-023-02380-0)
Supplement: Supplementary file 1 — Additional file 1. [file 12890_2023_2380_MOESM1_ESM.docx]

**Supplementary Table S1.** **Correlation of quantitative CT and spirometry parameters**

| parameters | FEV_1_ | FEV_1_ %pred | FVC | FVC %pred | FEV_1_/FVC | MMEF %pred | RV | TLC | RV/TLC |  |
| --- | --- | --- | --- | --- | --- | --- | --- | --- | --- | --- |
| PRM^fSAD^ | 0.096(0.274) | -0.042(0.636) | 0.13(0.140) | 0.068(0.443) | -0.183(0.037) | -0.309(<0.001) | 0.346(<0.001) | 0.232(0.008) | 0.258(0.003) |  |
| PRM^Emph^ | 0.196(0.025) | 0.130(0.138) | 0.271(0.002) | 0.182(0.037) | -0.196(0.025) | -0.240(0.006) | 0.359(<0.001) | 0.304(<0.001) | 0.214(0.014) |  |
| PRM^Normal^ | -0.179(0.041) | -0.054(0.541) | -0.244(0.005) | -0.148(0.092) | 0.201(0.022) | 0.308(<0.001) | -0.339(<0.001) | -0.267(0.002) | -0.209(0.017) |  |
| LA_5_ | 0.227(0.009) | 0.169(0.054) | 0.234(0.007) | 0.163(0.063) | -0.023(0.798) | 0.179(0.040) | 0.106(0.230) | 0.134(0.128) | -0.040(0.652) |  |
| LA_6_ | 0.289(0.001) | 0.357(<0.001) | 0.239(0.006) | 0.252(0.004) | 0.101(0.250) | 0.338(<0.001) | 0.112(0.205) | 0.156(0.075) | -0.089(0.313) |  |
| ***Abbreviations:*** FEV1, forced expiratory volume in 1 second; FVC, forced vital capacity; MMEF, maximal mid-expiratory flow; RV, residual volume; TCL, total lung capacity. LA: luminal area; PRM, parametric response mapping; PRM^Emph^, emphysema by PRM; RPM^fSAD^, functional small airway disease; PRM^Normal^, normal lung parenchyma by PRM. | | | | | | | | | | |
